# High-Resolution Ultrasonographic Anatomy of the Carpal Tendons of Sporting Border Collies

**DOI:** 10.3390/ani12162050

**Published:** 2022-08-11

**Authors:** Maria Grazia Entani, Alessio Franini, Gabriele Barella, Roberta Saleri, Fabio De Rensis, Giliola Spattini

**Affiliations:** 1 Sporty Dog, 25123 Brescia, Italy; 2 Clinica Veterinaria CMV, 21100 Varese, Italy; 3 Department of Veterinary Medical Science, Parma University, 43126 Parma, Italy; 4 Clinica Veterinaria Castellarano, 42014 Castellarano, Italy

**Keywords:** ultrasound, tendon, carpus, agility dogs, Border Collie

## Abstract

**Simple Summary:**

Dog agility is a canine sport that has gained popularity among pet owners in recent decades. Because of the high-performance level, injuries to dogs competing in this sport are becoming frequent. The need for better knowledge of the anatomy of the structures involved in athletic movements is an essential starting point for correctly managing agility-related injuries. The aim of this paper was to investigate the ultrasonographic anatomy of the carpal joint, creating a baseline reference for the Border Collie, which is the breed most utilised in agility competitions. The data acquired could be of use in future studies regarding sport-induced injuries in the canine carpus.

**Abstract:**

Recent literature has demonstrated that high-resolution ultrasonographic anatomy of the canine carpus is possible; however, only the structures of the dorsal face were described. The aims of this prospective study were: (1) to describe the normal ultrasonographic appearance of the carpal tendons in sporting Border Collies; (2) to measure the height, length, and thickness of the tendon at the radial ulnar notch level in order to create a baseline reference for the breed, and (3) to describe a standardised protocol to ultrasonographically evaluate the carpal faces and visible tendinous structures. A pilot study based on ten cadaveric front limbs was used to identify the structures. A subsequent clinical phase of the study using twenty-six Border Collies was recorded. The tendons of the *Extensor Carpi Radialis*, *Extensor Digitorum Communis*, and *Extensor Digitorum Lateralis* were identified and followed from the tenomuscular junction to the distal insertion on the dorsal face of the digits. On the lateral face, the tendon of the *Extensor Carpi Ulnaris* was recognised and followed. On the palmar face, the two heads of the *Flexor Carpi Ulnaris* tendon ending on the accessory carpal bone, the adjacent *Flexor Digitorum Superficialis* tendon, and the deep and medially located *Flexor Digitorum Profundus* tendon were seen and followed. The *Flexor Carpi Radialis* and the *Abductor Pollicis Longus* tendons were seen in the medial carpal face. The ulnar notch of the radius was used as the measurement and starting point of the ultrasonography. These data could be used as a standard reference in the case of chronic overuse and trauma-induced changes in the canine carpus.

## 1. Introduction

In human athletes, the ultrasonographic evaluation of an injured wrist is a reliable diagnostic tool associated with clinical examination, radiographic examination, and other advanced imaging techniques (e.g., magnetic resonance imaging) [1,2,3]. In veterinary medicine, ultrasound has been shown to help assess soft tissue injuries of several canine and equine front- and hindlimbs. One paper has been published regarding the ultrasonographic anatomy of the canine carpus; however, only the dorsal structures were investigated, and several breeds were studied together [4].

Canine sports are on the rise, and sport-related injuries have been increasingly noted. In a survey on Racing Greyhounds, the rate of carpal injuries reached 17%; in marathon sled dogs, shoulder and carpal injuries are commonly encountered [5,6]. In 2013, Cullen et al. provided data regarding the nature of the injuries in dogs involved in high-level agility competitions. They found that 7% of 1602 agility-related injuries were located in the carpus [7]. The workload on forelimbs during sports activities and the number of carpal injuries described in canine athletes suggest that the carpal joint can undergo significant load stress during the athletic gesture.

Canine bodyweight is unequally distributed between the forelimbs and the hindlimbs during unimpeded walking, with approximately 60% of the vertical force exerted on the forelimbs. When trotting, more than the dog’s entire body weight is carried by a single forelimb. It increases to over twice the body weight during a gallop. In fact, as reported in a previously published paper regarding ground reaction forces, a galloping Labrador Retriever sustains 2.6 times its body weight on the forelimbs while trotting at 27 km/h. Regarding agility activity, the literature suggests that more than 4.5 times body weight is borne by the forelimbs in hurdle jumps landing during agility tracks [8,9]. Diagnosing carpal injuries can be demanding, especially in sporting dogs; misdiagnoses can lead to the end of the dog’s career. The knowledge of ultrasound anatomy is mandatory for establishing a correct ultrasound diagnosis and subsequent treatment.

The aim of this study was to define the normal US anatomy of the carpus in Border Collies and create a standardised measurement reference in sporting patients of this breed to be used as a starting point in a clinical setting.

## 2. Materials and Methods

### 2.1. Pilot Study

According to previously published papers, a pilot study was carried out to identify and correlate the gross anatomy of the carpal tendons with their imaging appearance [4,10,11]. The final study comprised two phases: identification ex vivo and description in vivo. The study was conducted according to the Ethical Committee of Parma University (8/CESA/2022). Owner consent was obtained to collect the specimens and use the data included in the study.

**Figure 1:**
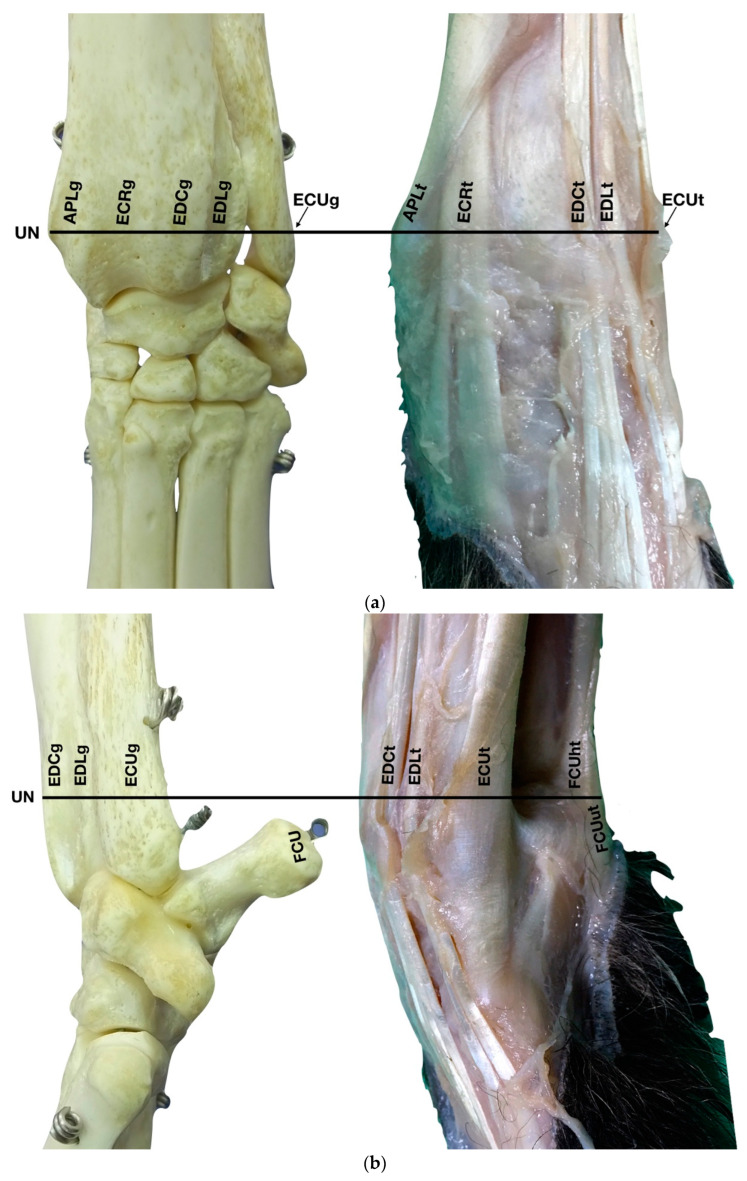

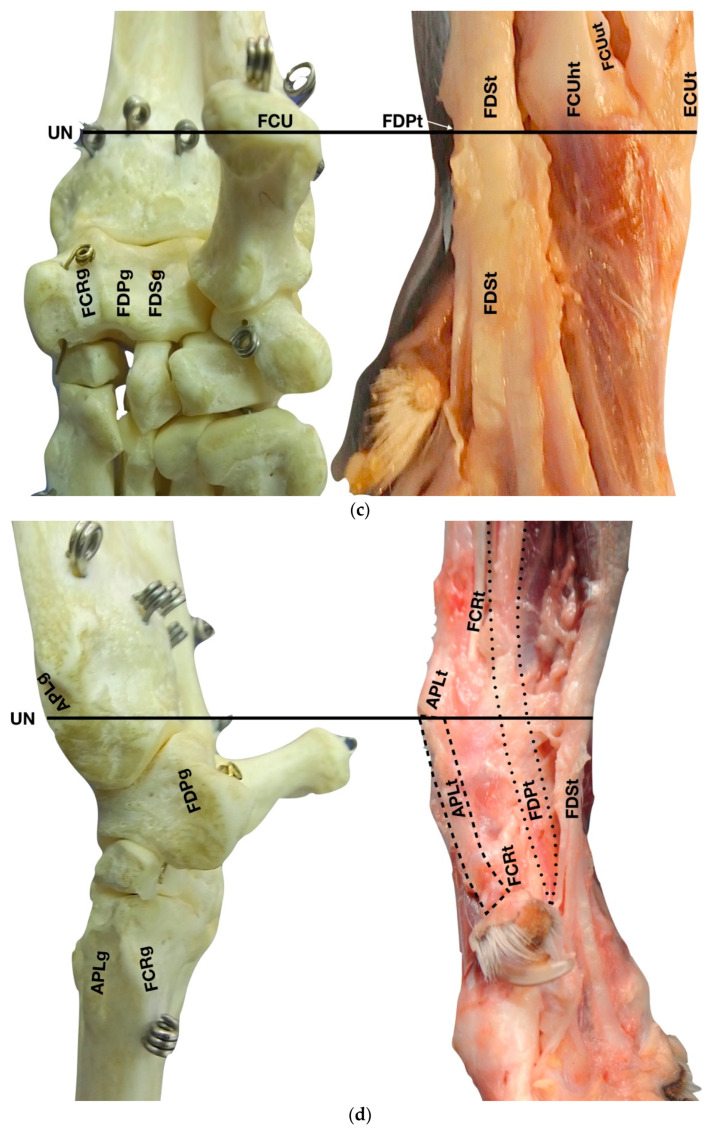

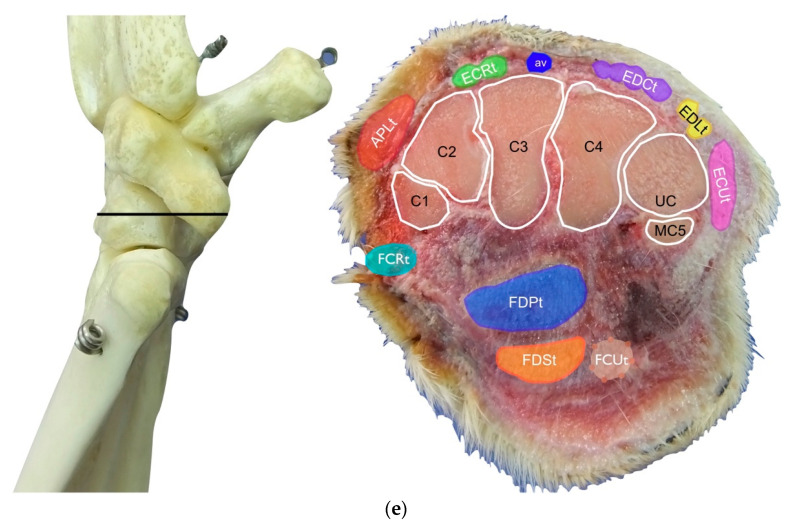
(**a**) Dorsal carpal face. The ulnar notch of the radius (UN) is palpated, and the tendon of the *Abductor Pollicis Longus* (APLt) and the adjacent *Extensor Carpi Radialis* (ECRt) are found. The grooves beneath are labelled in the bone specimen (APLg and ECRg). The tendon of the *Extensor Digitorum Communis* (EDCt) and the small *Extensor Digitorum Lateralis* (EDLt) are easily palpated and visualised on the dorsolateral face. The corresponding grooves are labelled in the bone specimen. (**b**) The large tendon of the *Extensor Carpi Ulnaris* (ECUt) is easily visualised on the lateral carpal face. (**c**) On the palmar carpal face, the two heads (humeral and ulnar) of the *Flexor Carpi Ulnaris* tendon (FCUt) are easily seen, ending at the accessory bone. On this specimen, part of the ulnar head of the *Flexor Carpi Ulnaris* tendon (FCUut) was removed to show the humeral head of the same tendon (FCUht). Medially, the tendon of the *Flexor Digitorum Superficialis* (FDSt) is visible and is hiding the deeper *Flexor Digitorum Profundus* (FDPt) (white arrow). The tendon grooves are labelled on the osseous specimen. (**d**) The medial carpal face. The FDSt has been displaced laterally to expose the FDP tendon beneath. The tendon of the small *Flexor Carpi Radialis* tendon (FCRt) is visible between the FDPt and the tendon of the APL, and (**e**) transverse section of the front limb at the level of the distal carpal row, ventral to the base of the accessory bone. The topographic location of the tendons is visible. In this image, the virtual location of the FCUt has been drawn to give an overview of the corresponding position of all the carpal tendons. However, in reality, the tendon ends proximally at the base of the accessory bone. av, accessory cephalic vein; C1, carpal bone one; C2, carpal bone two; C3, carpal bone three; C4, carpal bone four; UC, ulnar carpal bone; MC5, fifth metacarpal bone.

Ten carpi were obtained from the cadavers of five mixed breed dogs weighing from 13.1 to 22.9 Kg, which had died due to reasons unrelated to this study. They were divided into two groups: five carpi for performing an anatomic dissection and five for undertaking an ultrasonographic examination. No lameness had been reported in their clinical chart. Before the dissection, the carpal joints were radiographed and were reported to be within normal limits by a board-certified radiologist (GS). The radiographs were taken in standard dorso-palmar, and mediolateral projections; and varus and valgus instability were excluded by palpation [12]. The limbs were transected at the level of the distal humeri and were frozen at −18 °C. Three cadavers had both front limbs defrosted; for each of the three cadavers ultrasound identification was performed on one limb and anatomical dissection on the other simultaneously. In the remaining two cadavers only one limb was defrosted and scanned while the others, still frozen, were sectioned transversely using a band saw with a three-millimetre gap. Photographs of the anatomic specimens were taken for subsequent comparison and correlation with corresponding ultrasonographic images (Figure 1a–e).

For the anatomic identification of the carpal structures, the Nomina Anatomica Veterinaria and Miller’s Anatomy of the Dog were used as references [13,14].

### 2.2. Ultrasonographic Evaluation

Twenty-six sporting Border Collies presented at the Castellarano Veterinary Clinic from 1 January 2017 to 31 January 2018 were enrolled in this study. They were all highly trained agility dogs presented for forelimb lameness after a trauma sustained during training or for decreased sport performance. The relevant injuries were not located in the carpus in any of the patients. The inclusion criteria for the study were (1) the absence of pain or reduction in the carpal range of motion during the physical examination; (2) the lack of radiographic changes on carpal radiographic assessment; and (3) no evident lesions detected on the carpal ultrasonographic examination. Twelve carpal joints were excluded from the study due to carpal pain on physical examination rather than radiographic or ultrasonographic changes. A total of 40 carpal joints were available for this study. 

The patients were standing on the table with the side of the limb to be examined close to the US machine and the operator. The patient’s limb was clipped from the mid-length of the radius and ulna to the metacarpophalangeal region. A solution of medical alcohol was applied to scrub the skin, and abundant warmed acoustic gel was applied to both legs five to fifteen minutes before starting the scanning examination. 

The warmed gel was essential for increasing the ultrasound passage through the skin of the carpus and for increasing image quality [15]. The sonographic examination was performed by the same operator (GS) using an 8–18 MHz golf stick probe (Logiq S8, GE). Standoff pads or sedation were not needed. On the transverse scan, the medial portion of the patient was displayed to the left of the ultrasound screen and the lateral to the right (from the ultrasonographer’s point of view facing the screen). On the longitudinal scan, the proximal portion of the patient was displayed to the left of the ultrasound screen and the distal to the right (from the ultrasonographer’s point of view facing the screen). Both limbs were routinely evaluated. The ultrasonographic assessment was part of the routine evaluation for front limb lameness in highly performing agility patients. 

### 2.3. Statistics

All the data collected were used for the statistical analysis. The median value and standard deviation (SD) were calculated for age, weight, ECR (right and left), EDC (right and left), EDLt (right and left), ECUt (right and left), FCUt (right and left), FDSt (right and left), FDPt (right and left), FCRt (right and left), and APLt (right and left).

All the measurements concerning tendons were assessed for linear correlation (r-value) in relation to age and weight. A value of r ≥ 0.8 was considered a good correlation. Student’s *t*-test was used to assess the presence of statistically significant differences between the left and right side of the same anatomical structure (i.e., EDC right compared to EDC left). A value of *p* < 0.05 was considered to be statistically significant.

## 3. Results

During the pilot study, ten carpi were obtained from the cadavers of five medium-sized mixed breed dogs of which three were female (60%), and two were male (40%). The dogs’ ages ranged from thirty to one hundred five months, with a median age of sixty-seven months. The weight ranged from 13.1 to 22.9 kg with a mean of 16.4 kg.

Twenty-six clinically forelimb lame Border Collies were selected for the second part of the study. There were eleven intact males (42%), four neutered males (16%), and eleven spayed females (42%). Age ranged from thirty-two months to one hundred thirty-three months, with a mean age of seventy-three months. Their weight ranged from 15.4 to 21.0 kg with a mean of 17.3 kg. Patient data, relevant lesions, and tendon measurements at the ulnar notch are summarised in Table S1 (Supplementary Materials). The mean tendon height, length, and thickness together with the standard deviations are summarised in Table 1.

No statistically significant linear correlation was found between age and the thickness of the tendons. The measurements belonging to right ECR l (length), right EDC t (thickness), left EDL h (height), right EDL h, right FDP h, right FCR h, right APL h, left EDC t, left ECU t, and left FDP t had a positive linear correlation with weight. As the weight increased, the tendon measurement increased.

Statistically significant differences were found in the thickness of the left ECR and EDC tendons. These tendons were statistically significantly thicker on the left side than on the right side of the same patient (*p* < 0.05).

### 3.1. Dorsal Carpal Face

#### 3.1.1. *Extensor Carpi Radialis* Tendon (ECRt) Ultrasonographic Anatomy

**Figure 2:**
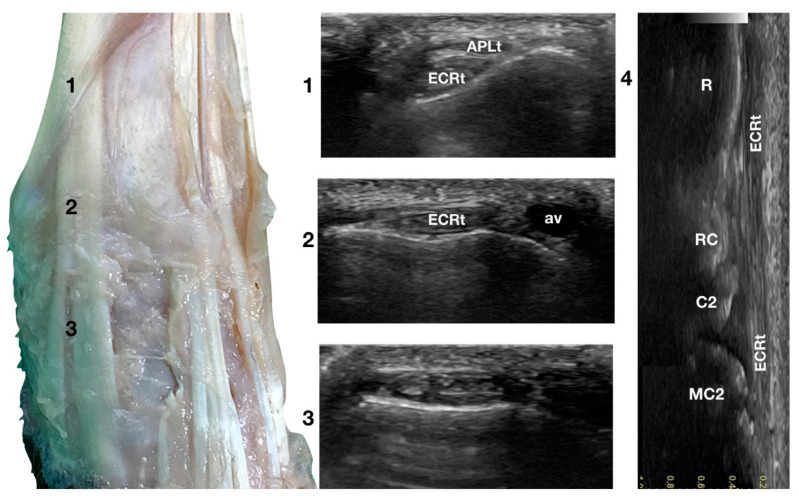
Dorsal carpal face centred on the *Extensor Carpi Radialis* tendon. At point 1, the APL superficial and thin emerging tendon covers the thick tendon of the ECR. At the ulnar notch of the radius (2), the ECR tendon is easily recognised due to the thick hyperechoic layer that defines its contour (peritendineum). Medial to the tendon, the accessory cephalic vein (av) is visible. At the intercarpal joint (3), the tendon subdivides into the smaller medial part and the more significant lateral part, as is easily seen in the image. However, they are still enclosed in the same tendon sheath that completely divides into two tendons distally. Image 4 is the visualisation of the longitudinal image of the medial portion of the ECRt. The bony surface beneath is recognisable. R, radius; RC, radiocarpal bone; C2, carpal bone two; MC2, second metacarpal bone.

The ulnar notch of the radius was palpated with the thumb and used as a starting point to assess the dorsal structures of the carpus (Figure 1a). The tendons of all the faces were measured at this level in transverse and longitudinal scan planes. Immediately dorsally to the easily recognisable ulnar notch, the *Abductor Pollicis Longus* tendon (APLt) (only minimally perceptible) was palpated. The adjacent, more dorsal, more profound and easier to recognise groove of the *Extensor Carpi Radialis* tendon (ECRt) was felt immediately after. At this level, the ECRt was thick, round, and minimally movable, and an easy start for the systematic carpal evaluation. The probe was in a transverse scan plane, starting at the ulnar notch level. The tendon was ultrasonographically well defined by the surrounding tissues due to the thin hyperechoic line, which enhanced the contour (peritendineum) and the minimal fluid accumulation in the surrounding fibrous tendon sheath (Figure 2).

Sliding the probe dorsally, the ECRt became thinner and closer to the bone, and the APLt and its tenomuscular junction were seen more superficially, obliquely crossing the path of the straight ECRt. At the ulnar notch level, the ECRt thickness reached the maximum, and was separated from the skin and the periosteal surface of the radius by a thick fascia and fat layer. Just distal to this level, the tendon flattened again and became wider, quickly assuming a horizontal figure eight shape, and subdividing into two parts: a thin medial tendon and a more prominent lateral tendon. At the intercarpal joint level, the two tendons split. The medial tendon (*Extensor Carpi Radialis Longus*) showed a narrow and rounded shape. It became thinner and less distinct distally until it ended at the base of the second metacarpal bone. This tendon was easier to follow distally when imaged on longitudinal view, obtained by rotating the probe clockwise by 90 degrees to have the proximal portion of the tendon to the left of the screen. The lateral part of the tendon (*Extensor Carpi Radialis Brevis*) was oval, flattened, and ended on the base of the third metacarpal bone. This tendon was easily followed distally in the longitudinal scan plane.

The tendons showed a linear, dense, and hyperechoic fibrillar pattern in the transverse and longitudinal planes. The two tendons showed a progressive reduction in their diameters; however, they broadened distally at the insertions. Carpometacarpal flexion aided the examination and allowed checking whether adhesion or subluxation of the tendon rather than deeper joint effusion or bone surface remodelling was present. Minimal synovial effusion (less than 1 mm in thickness) was often but not always seen in the radiocarpal and intercarpal joints and, less commonly, in the carpometacarpal joint. Lateral and superficial to the ECRt, the accessory cephalic vein was seen. The position of the vein could vary if too much pressure was applied to the probe.

#### 3.1.2. *Extensor Digitorum Communis* Tendon Ultrasonographic Anatomy (EDCt)

**Figure 3:**
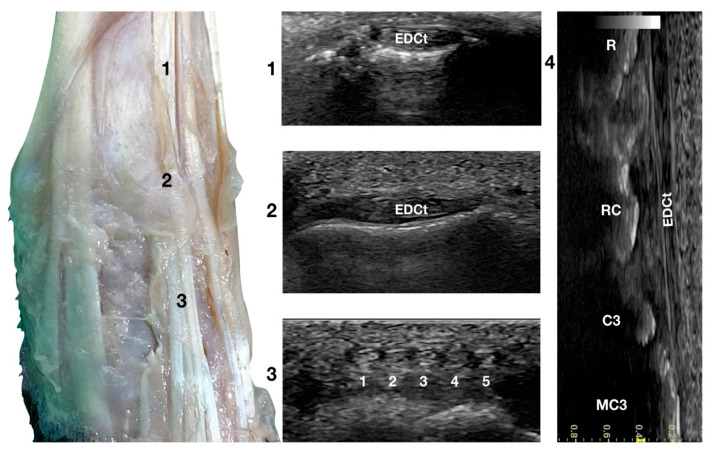
Dorsal carpal face centred on the *Extensor Digitorum Communis* tendon. At the ulnar notch level (2), the EDC tendon is thin and large, easily differentiated from the ECRt by its shape and position. Dorsally, just distal to the tenomuscular junction (1), the tendon is thicker and narrower. At the level of the radiocarpal joint (3), the tendon subdivides into four small tendons (white 2 to 5). The small, medial tendon of the *Extensor Digiti I Longus* is often visible (white 1). On the longitudinal scan plane (4), the fine fibrillar pattern of the thin tendon is visible. The image shows the part of the tendon that crosses the radius (R), the radiocarpal bone (RC), the carpal bone three (C3), and the third metacarpal bone (MC3).

Just lateral to the groove of the *Extensor Carpi Radialis* tendon and lateral to the large accessory cephalic vein, in the more prominent but shallower *Extensor Digitorum Communis* groove, on the dorsal surface of the carpus, the *Extensor Digitorum Communis* tendon (EDCt) was palpated and easily visualised in a transverse scan plane (Figure 1a). At the ulnar notch level, the tendon is thin and flattened with a markedly oval shape and is easily recognised by the medially located, rounder, *Extensor Carpi Radialis* tendon (Figure 3).

In a transverse scan plane, sliding the probe proximally, the EDCt became rounder and thicker at the tenomuscular junction. Sliding the probe distally, the EDCt was enclosed in a common synovial tendon sheath and passed through the lateral distal sulcus of the radius. The *Extensor Retinaculum* was seen as a thick hyperechoic line. Most of the time, this structure was ultrasonographically indistinct from the tendon sheath. The *Extensor Retinaculum* surrounded the tendon and blended with the periosteum on the lip of the carpal articular surface. At the level of the radiocarpal joint, the uniformly flattened EDCt divided into four separate thin tendons. They passed on the dorsal surface of the corresponding digits, ending in the dorsal portion of the ungual crest of the distal phalanges. With the 18 MHz probe, it was possible to differentiate the small tendons clamped together from the intercarpal joints and which became more distinct structures when progressing distally.

The tendon of the *Extensor Digiti I Longus* was inconsistently seen medially. In this case, five small tendons were seen running distally on the dorsal surfaces of the metacarpal bones. The tendon thickness reduced sharply at the subdivision, and the distal portion of the tendons was often too thin to be visible on the transverse plane. At the same time, it was possible to see its termination on the longitudinal scan planes when the patient’s paw was elevated from the ground and the digits were extended. The tendons were thin, having a straight path and the typical fibrillar pattern. The extension and flexion of the digits helped to define the tendon insertion and assess tendon function, lesions, adhesion, and subluxation. 

#### 3.1.3. *Extensor Digitorum Lateralis* Tendon (EDLt) Ultrasonographic Anatomy

**Figure 4:**
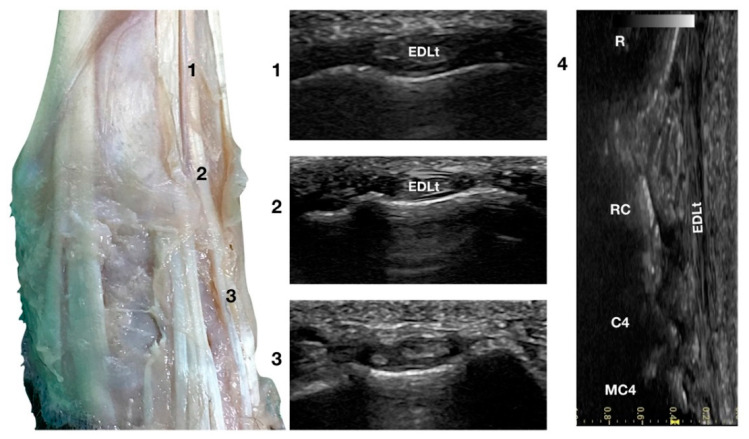
Dorsal carpal face centred on the *Extensor Digitorum Lateralis* tendon. At the ulnar notch level (2), the EDLt is seen as a thin oval tendon embedded in the deep *Extensor Lateralis* groove. Proximally (1), the tendon is thicker. The EDLt divides into two parts distally at the intercarpal joint (3). On the longitudinal scan plane (4), the fine fibrillar pattern of the thin medial tendon is visible. The bony surface beneath is recognisable. R, radius; RC, radiocarpal bone; C4, carpal bone four; MC4, fourth metacarpal bone.

Just lateral to the EDCt at the ulnar notch of the radius, on a transverse scan, the *Extensor Digitorum Lateralis* tendon (EDLt) was seen as a small, oval to slightly rounded shaped structure (Figure 1a). It was more dorsomedially located than the more prominent *Extensor Carpi Ulnaris* tendon (ECUt), easily palpated on the lateral face of the carpus. It was the only tendinous structure lateral to the EDCt and medial to the ECUt. Proximally, the tendon maintained the same size and shape until the tenomuscular junction. Distally, at the intercarpal joint level, the homogeneous echogenicity of the tendon was interrupted by an oblique hyperechoic line that demarcated the two components arising from the united tendon (Figure 4).

The two parts of the EDLt passed through the groove between the distal ends of the radius and the ulna, over the dorsolateral border of the carpus to the metacarpus, and then diverged from each other. The two thin tendons (the medial is the smaller) passed over the dorsolateral surface of the corresponding metacarpal bones (third and fourth for the medial tendon, and the ulnar carpal bone for the lateral tendon). They ended on the dorsal surface to the distal phalanges of digits III, IV (the medial tendon divided into two branches), and V (the lateral branch). They were surrounded by a thick hyperechoic line (fibrous tendon sheath). The tendons, even if thin, were seen in the longitudinal scan until the mid-metacarpal diaphysis. It is possible that, in larger breed dogs, the tendon can be evaluated more distally. Elevating the foot from the ground and rotating the probe to check the longitudinal scan plane often helped in examining the distal portion. The extension and flexion of the lateral digits helped to better differentiate the tendon from the surrounding tissues and assess tendon function, lesions, adhesion, and subluxation. 

### 3.2. Lateral Carpal Face

#### *Extensor Carpi Ulnaris* Tendon Ultrasonographic Anatomy (ECUt)

**Figure 5:**
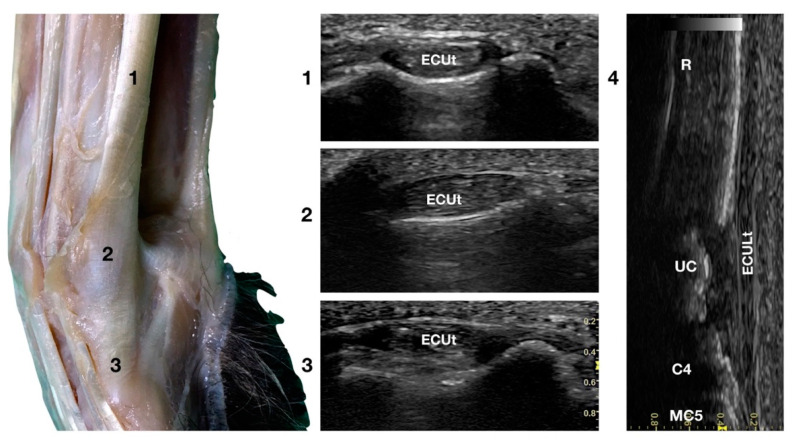
Lateral carpal face centred on the *Extensor Carpi Ulnaris* tendon. At the ulnar notch level (2), the *ECUt* is seen as a wide and flattened oval tendon in direct contact with the osseous surface. Proximally (1), the tendon is thick and narrow. The ECUt runs on the lateral face of the carpus and ends at the base of the fifth metacarpal bone where it reaches its maximum width (3). On the longitudinal scan plane (4), the prominent fibrillar pattern of the large tendon is visible. The osseous surface beneath is recognisable. R, radius; RC, radiocarpal bone; C4, fourth carpal bone; MC5, fifth metacarpal bone.

The large *Extensor Carpi Ulnaris* tendon (ECUt), also known as *Ulnaris Lateralis* tendon was easily palpated and recognised as the most prominent structure on the lateral surface of the carpus (Figure 1b). When palpated, the tendon was firm in a standing patient, resembling a bony structure. This was due to the tendon fibrillar density and to the fact that the tendon ran in close contact with the underlying bony surface. At the ulnar notch level, the tendon was recognised in the transverse plane, as quite a flattened and relatively hypoechoic structure, rich in thin hyperechoic foci, and surrounded by a hyperechoic line (peritendineum) (Figure 5).

In the larger patients, a multilayer onion-like appearance was rarely evident; the tendon was more uniform and homogeneous in the smaller patients. Proximally, reaching the tenomuscolar junction, which was located in the middle region of the antebrachium, the tendon was thicker and less wide. From its origin, the broad tendon passed laterally over the carpus, being held in place by a thin, transverse, tendinous band, which attached the ECUt to the accessory carpal bone. The tendon showed a prominent overall hyperechogenicity with a dense fibrillar pattern in the longitudinal scan. The ECULt ended laterally at the base of metacarpal V, and widened toward its insertion, achieving its maximum size. The standing position optimized the fibrillar pattern visualisation due to optimal alignment on the longitudinal scan. Carpometacarpal flexion and extension, and elevation of the fifth digit were used to check the tendon for adhesion or reduced range of motion. Medial and lateral stress was assessed to check for tendon compliance. The dynamic examination was easier to carry out when the paw was elevated.

### 3.3. Palmar Carpal Face

#### 3.3.1. *Flexor Carpi Ulnaris* Tendon Ultrasonographic Anatomy (FCUt)

**Figure 6:**
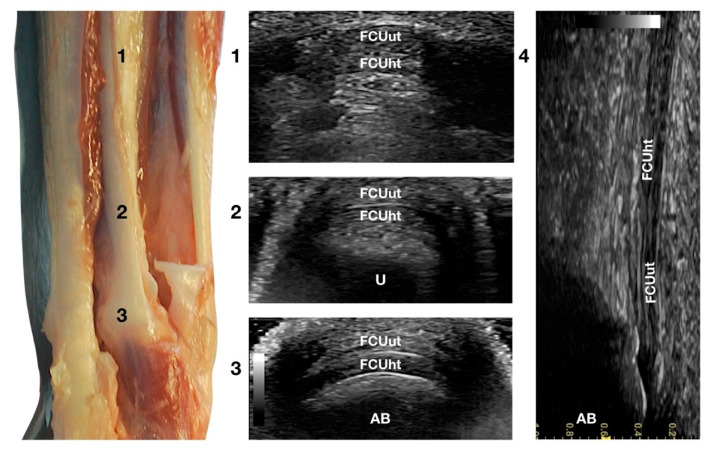
Palmar carpal face centred on the *Flexor Carpi Ulnaris* tendon. Part of the distal attachment of the FCUut covering the FCUht was removed to show the thicker, deeper portion. At the ulnar notch level (2), the FCUt is a broad and flattened elliptical tendon subdivided into two nearly equal parts by a thin hyperechoic line. Some fat separates the tendon from the deeper osseous contour of the distal portion of the ulna (U). Proximally, at the tenomuscular junction (1), the combination of the two-part forms a rounder and narrower tendon. The tendon becomes thinner and more expansive due to the insertion based on the accessory carpal bone (AB) (3). On the longitudinal scan plane (4), the prominent fibrillar pattern of the tendon components is visible.

The two tendinous portions of the *Flexor Carpi Ulnaris* tendon (FCUt) were visible and easily palpated at their attachment to the accessory carpal bone (Figure 1c). A longitudinal approach starting distally to the tendinous bone attachment, easily palpated, and progressing proximally up to the tenomuscular junctions, made the identification of these two structures easy (Figure 6).

The ulnar head of the *Flexor Carpi Ulnaris* tendon (FCUut) appeared as a thin hyperechoic band that encroached on the thicker humeral head tendon (FCUht). They ended in a close relationship at the proximal aspect of the accessory carpal bone. The two tendinous parts were more clearly identified on the transverse scan plan. The combined tendons appeared as a flat elliptical hyperechoic structure, divided into two nearly equal bands by a thin hyperechoic line. Proximally, the united tendon was thicker and less wide; when extending distally, the tendon became elliptical and finally oval with a decreased thickness at the attachment site on the top of the accessory bone.

Flexing and extending the carpus, and stressed mediolateral movements could be used to evaluate subluxation, partial avulsion, adhesion, and reduced range of motion. The dynamic evaluation was more complete with the paw in elevation.

#### 3.3.2. *Flexor Digitorum Superficialis* Tendon Ultrasonographic Anatomy (FDSt)

**Figure 7:**
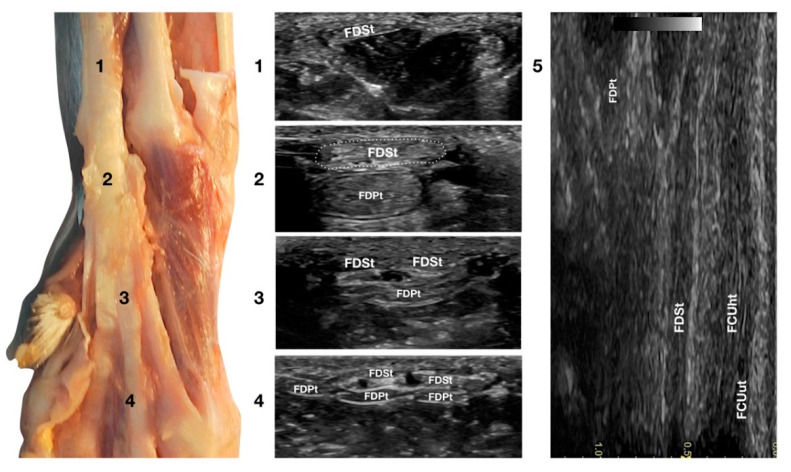
Palmar carpal face centred on the *Flexor Digitorum Superficialis* tendon. At the ulnar notch level (2), the FDSt is a broad and flattened elliptical structure surrounded by echoic ill-defined tissue (the *Retinaculum*). It is just superficial to the larger *Flexor Digitorum Profundus* tendon. Proximally, the tendon is very superficial and flat at the tenomuscular junction (1). Distally, in the proximal third of the metacarpus (3), the tendon starts to divide into four structures that diverge and follow the more profound and larger parts of the FDPt (4). On the longitudinal scan (5), obtained by putting the probe in the hollow medially to the accessory carpal bone and angling the probe slightly laterally, the fine fibrillar pattern of the FDSt is seen parallel and separated from the more lateral and superficial FCUt by fat and the *Retinaculum*. The deeper FDPt is barely visible since it is not perfectly parallel to the other two tendons. The FDPt starts deeper and runs obliquely to become relatively superficial at the accessory bone passage where it pairs with the FDSt until their termination.

Just medial to the accessory bone, so close that in a longitudinal scan plane, a minimal medial angulation of the probe gave the impression of some degree of continuity with the FCUt; the Flexor Digitorum Superficialis tendon (FDSt) was seen parallel to the FCUt but extending distally to the accessory carpal bone (Figure 1c). On transverse images, moving the probe slightly medially to the accessory bone, the FDSt was seen as a large, echoic, elliptical structure, approximately twice as wide as thick. The Flexor Retinaculum was a thick connective layer that created a nearly uniform echoic ring surrounding the tendon and separating its deeper margin from the Flexor Digitorum Profundus tendon (FDPt) beneath (Figure 7).

Progressing proximally, the tendon quickly became thinner and broader, assumed a superficial position, and was separated from the FDPt by several muscle bellies. Just distal to the accessory bone, the tendon thickened and paired with the deeper FDPt. In the proximal third of the metacarpus, the FDSt flattened, becoming progressively thinner and remaining superficial to the thicker FDPt. At this level, they split into four parts that diverged from the second to the fifth metacarpophalangeal joint. At this level, the paw partially interferes with the visibility of such small structures. By elevating the foot and using the palmar aspect of the digit with a golf stick probe, it was possible to have an acoustic window distal to the paw pad. This small window often made it possible to recognise it in a transverse scan, a small elliptical structure corresponding to the fusion of the FDS and FDP tendons. A rich fibrillar tendinous pattern was noted in the longitudinal scan of the tendon. The phalangeal termination was often best visualised in the longitudinal scan plane using the palmar digital surface, and a single thin tendon, representing the combination of the FDS and the FDP tendons, was sometimes recognised and rarely followed up to the FDSt insertion at the palmar surface of the base of the middle phalanx. Flexing and extending the digits and the carpus, and stressing the carpus and the digits mediolaterally enhanced the range of motion and the degree of tendon compliance, and tested the *Extensor Retinaculum* function.

#### 3.3.3. *Flexor Digitorum Profundus* Tendon Ultrasonographic Anatomy (FDPt)

**Figure 8:**
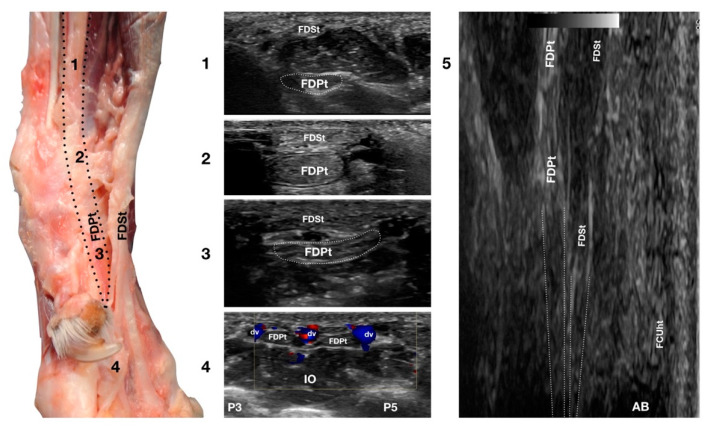
Palmaromedial carpal face centered on the *Flexor Digitorum Profundus* tendon. The FDSt were displaced to show the FDPt beneath. At the ulnar notch level (2), the FDPt is a wide, thick, oval structure, surrounded by ill-defined echoic tissue (the *Retinaculum*). It is just a little deeper but is in close contact with the thinner *Flexor Digitorum Superficialis* tendon. Proximally, at the tenomuscular junction (1), the tendon is located in a deep position. It runs obliquely distally, becoming progressively more superficial until the level of the radial notch where it pairs with the FDSt (2). At the level of the proximal third of the metacarpus, the tendon becomes thinner and wider (3) until it starts to divide into four parts that diverge and follow the superficial and thinner parts of the FDSt (4). The thin tendons of the FDS and of the FDP are outlined by the small branches of the digital veins (dv). The thick interosseous muscles (IO) separate the tendons from the third (P3), fourth (P4), and fifth (P5) metacarpal bones. On the longitudinal scan (5) obtained by putting the probe in the hollow area medial to the accessory bone and angling the probe slightly medially, the fine fibrillar pattern of the FDPt is seen, converging toward the FDSt. The more superficial FCUt and the accessory bone (AC) are barely visible.

In the hollow between the protruding portion of the accessory carpal bone and the caudal margin of the radial ulnar notch, in a transverse scan plane, the more superficial and elliptical FDSt and the deeper, larger and rounder *Flexor Digitorum Profundus* tendon (FDPt) were easily seen as paired structures (Figure 1d). Using an adequate amount of gel helped to align the probe with an area having an uneven surface. The thick tendon was grooved on the palmar surface of the carpus that was converted into the carpal canal by the *Flexor Retinaculum* surrounding the FDS and the FDP tendons (Figure 8).

Proximally, the FDPt ran obliquely and had a deep position, relatively closed to the caudal margin of the radius and ulna. The obliquity of the tendon with respect to the surface of the skin created the false impression of a relatively hypoechoic tendon, due to an anisotropy artefact. The tendon became parallel to the skin at the level of the accessory carpal bone and, at this level, it appeared as a large, oval, echoic structure with a rich fibrillar pattern. On the proximal portion of the metacarpus, the FDP tendon divided into four branches from digits II to V and ran distally, covered by the corresponding branches of the FDS tendon. The technique for checking the distal aspect of the tendon was the same as that previously reported for the FDSt. While the FDSt inserted into the palmar surface at the base of the middle phalanx, the FDPt inserted into the tuberosities of the distal phalanges of digits II to V. This part was only visible if accurate preparation of the palmar pad of the digits was carried out. Abundant gel was applied to an elevated pad for at least five minutes before the scanning. In some patients, the dryness and thickness of the palmar pad prevented imaging of this region. On the longitudinal scan, a dense fibrillar pattern was recognised in the proximal portion of the tendon while, in the most distal and thinner portion, the tendon appeared relatively hypoechoic and uniform, especially when the tendon thickness was less than 1 mm. The oblique path of the tendon emerging from a deep position to be parallel to the FDSt was better assessed on the longitudinal plane. The flexion and extension of the carpus and the digits, together with the mediolateral stressed poses, were used to assess adhesions, range of motion, and subluxations of the tendon.

### 3.4. Medial Carpal Face

#### 3.4.1. *Flexor Carpi Radialis* Tendon Ultrasonographic Anatomy (FCRt)

**Figure 9:**
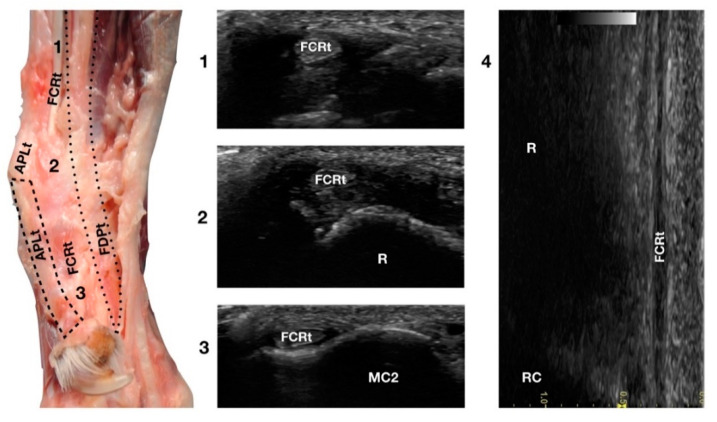
Palmaromedial carpal face centred on the *Flexor Carpi Radialis* (FCR) tendon. At the ulnar notch (2), the FCRt is a thin, relatively hypoechoic, oval structure surrounded by ill-defined and hypoechoic muscle fibres. Proximally, at the tenomuscular junction (1), the tendon is relatively superficial and better defined from the surrounding hypoechoic muscles. The radial surface is visible (R). At the level of the carpometacarpal joints, the tendon splits into two equal parts and becomes closer to the osseous surface of the base of metacarpal bone 2 or 3 (in this case it is MC2) where it terminates (3). On the longitudinal scan (4) obtained by aligning the probe to the palmar margin of the ulnar notch, the thin FCRt is seen as a hypoechoic structure with few visible fibrillar lines. The radial (R) and radiocarpal (RC) surfaces beneath are visible.

The *Flexor Carpi Radialis* was the thin tendon just palmar to the ulnar notch of the radius. It was difficult to palpate due to its small size; however, it was easy to find on ultrasound as it was the only tendinous structure in this region and was equally distanced from the APLt (medially located) and the FDPt (plantar). The FCRt was seen in the transverse scan as a small, oval to slightly rounded structure, homogeneous, and hypoechoic compared to the adjacent, more prominent tendons (FDPt and APLt). The FCR muscle lay in the medial part of the antebrachium directly under the skin and the antebrachial fascia. It covered the *Flexor Digitorum Profundus* muscle and appeared medial to the FDSt (Figure 1d). The thick fusiform belly merged into a flat tendon near the middle of the radius. At the flexor surface of the carpus, the FCRt ran through the medial aspect of the carpal canal enclosed in the *Flexor Retinaculum*, where a synovial sheath surrounded it, located superficially and medially to the FDP tendon (Figure 9).

At the carpometacarpal joint, it split into two distinct tendons that terminated on the palmar side of the base of metacarpals II and III, very close to the proximal articular surface. On longitudinal scan, it was a thin hypoechoic tendon (due to its size), seen medially to the FDPt and palmar to the APLt. The flexion and extension of the carpus and the digits and mediolateral stressed poses were used to assess the adhesions, range of motion, and subluxations of the tendon.

#### 3.4.2.* Abductor Pollicis Longus* Tendon Ultrasonographic Anatomy (APLt)

The ulnar notch of the radius was palpated with the thumb and used to recognise the *Abductor Pollicis Longus* tendon (APLt) lodged inside the groove delimited by the ulnar notch. At this level, the proximal oblique portion, enclosed in the *Abductor Pollicis* radial groove, emerged and changed the direction of its path, becoming parallel to the medial articular surfaces of the radiocarpal, intercarpal, and carpometacarpal joints, terminating at the base of the first metacarpal bone (Figure 1a,d).

**Figure 10:**
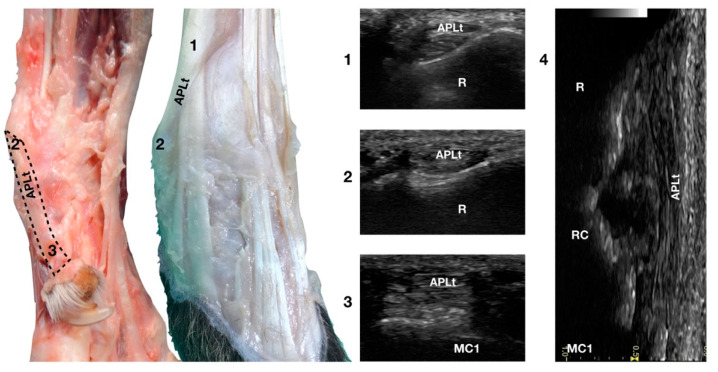
Palmaromedial and dorsal carpal faces. The oblique (1–2) and longitudinal (2–3) portions of the Abductor Pollicis Longus tendon are displayed. At the ulnar notch (2), after exiting the medial radial sulcus or the APL groove, the APLt changes inclination. Proximally, the APL follows the groove until the tenomuscular junction (1) crosses the ECRt beneath. At the ulnar notch, the oblique portion changes direction and exits the APL groove to become parallel to the medial surface of the carpus. It terminated at the base of the first metacarpal bone (MC1). On the longitudinal scan (4) obtained by aligning the probe to the medial margin of the ulnar notch, the thick APLt is seen as an undulating band rich in hyperechoic fibrillar lines. The radial (R), radiocarpal (RC), and first metacarpal (MC1) surfaces beneath are visible.

The *Abductor Pollicis Longus* muscle originated on the lateral surface of the radius, ulna, and interosseous membrane. Its fibres blended into a strong tendon toward the carpus, obliquely crossing the deeper ECRt and passing into the medial sulcus of the radius under the short medial collateral ligament (Figure 10).

On a transverse scan, the tendon appeared as a relatively rounded, small tendon with homogeneous echogenicity and a fine fibrillar pattern at the ulnar notch of the radius. A tendon sheath surrounded this segment, creating a thin hypoechoic halo delimited by a thin hyperechoic line. The proximal oblique portion was often more difficult to visualise due to the narrow acoustic window provided by the abductor pollicis groove in which the osseous surface appeared as a U-shaped hyperechogenic line with acoustic shadow. Proximally, at the tendomuscular junction, the tendon was seen as a flat, hypoechoic structure, superficial to the ECRt. Distally, the well-defined tendon was separated from the osseous surfaces by a hypoechoic, homogeneous band corresponding to the *Extensor Retinaculum*. The tendon inserted medially on the proximal aspect of the first metacarpal bone with an embedded sesamoid bone, not always mineralised or present. On a longitudinal scan plane, the best starting point was the distal margin of the ulnar notch from which the tendon of the APL emerged nearly parallel to the medial surface of the carpal rows and appeared as a well-defined band with a parallel and uniformly hyperechoic fibrillar pattern. The APLt was the only one not seen to be stretched when the patient was in a standing position; a slightly wavy path was noted. At the ulnar notch, the ultrasound probe was aligned with the APL groove to follow the tendon proximally until the tendomuscular junction. The first digit was flexed and extended during the ultrasound examination to evaluate the sliding motion of the abductor pollicis longus tendon. Stressed mediolateral rotations were used to check the range of motion and adhesion or subluxation [16,17]. 

## 4. Discussion

Tendons are mainly composed of bundles of collagen fibres running parallel to each other, which interweave and interconnect [18,19]. These bundles are large enough to be visible to the naked eye and are detectable when the US is performed on the long axis of the tendon with the incident sound beam perpendicular to it. If the probe is perfectly perpendicular to the tendon imaged, the entire echo arrives at the probe and can contribute to image formation. If the probe and the tendon to be examined are oblique, the major part of the reflected echo could escape the probe, resulting in hypoechoic focal regions, due to an anisotropy artefact that could be mistaken for tendon lesions. Anisotropy is one of the most often encountered artefacts, profoundly affecting the image quality of musculoskeletal ultrasound [20,21].

Tendons of the canine carpal joint tend to be perfectly parallel to the bones (except for the proximal portion of the *Abductor Pollicis Longus* tendon that runs obliquely) and are therefore ideal structures to be imaged using ultrasound. The number of linear reflecting lines within the tendons increases with the frequency of the transducer, and high-frequency transducers have improved the efficacy of defining small tendon lesions and typical anatomical structures. The cross-sectional profile of the above tendons is round to oval, homogeneous, and uniformly hyperechoic to the surrounding muscle. The echogenicity of the tendon is strictly correlated with the diameter, with the smaller tendon being more hypoechoic than the larger structures. With the high-frequency probe, it is also nearly always possible to visualise the tendon sheath surrounding the major part of the tendon in the region of the canine carpus [4]. 

In human gymnasts, the wrist is a highly overused and injury-prone region that requires repetitive extension and flexion, often coupled with weight-bearing [3]. Similarly, in canine athletes, weight-bearing during high-energy non-steady-state activities is considered to be one of the most important causes of carpal injuries [22,23]. 

The distal insertion of the *Extensor Carpi Radialis* tendon in racing Greyhounds was the first canine tendinous carpal structure described using ultrasound [24]. According to Williams et al., its role in carpal biomechanical analysis showed how the ECRt was a powerful stabiliser of the carpal joint during extension. No studies are available regarding agility Border Collies; however, the authors hypothesised that the principles studied and applied to racing Greyhounds could likely be applied to another canine sprinter.

In human athletes, overuse injuries of the *Extensor Digitorum Communis* tendon are not commonly described whereas traumatic injuries are frequently seen in contact sports (boxing or martial arts). The diagnosis is often clinical as the more common lesion is a sagittal injury at the distal side of the tendon, which leads to clear tendon luxation. Ultrasound is still routinely used for studying the extent of the lesion for treatment planning [25]. The EDCt is one of the dog’s longest tendons in the forearm, with a small cross-sectional area and the greatest amount of passive length changes. It has been highly recognised that its function is to achieve stabilisation opposing the hyperextension occurring at the carpus while experiencing high-speed forces (e.g., during high-speed locomotion or jump landing). The occurrence of primary extensor injuries in dogs, such as complete ruptures or impact lesions during sports activities, should be considered, although they have not been reported in the literature. 

The applied statistical analysis found that the thickness of the ECR and EDC tendons was increased on the left front limb as compared with the right. It is unclear why this happens; no data were found in the literature. Experienced dog handlers suggested that since most dog conductors are right-handed, they tend to favour their right side, forcing the dog to use more the left side. However, this has not been proven, and experienced dog handlers tend to be very careful to have a symmetric workload.

The *Extensor Digitorum Lateralis* muscle is one of the tightest muscles in dogs’ forelimbs and, in racing Greyhounds, this tendon seems to be involved in stabilising mechanisms. The literature has reported that it acts as a damper attenuating vibration within the limb similar to the equine *Flexor Digitorum Superficialis* [24]. The anatomic characteristics of its tendon suggest that is involved in elastic energy storage; however, its anatomical location on the dorsolateral aspect of the limb should suggest that this structure has a shock absorber function in preventing hyperflexion of the carpus. Agility overuse-related injuries have not been reported. 

In human athletes, the *Extensor Carpi Ulnaris* tendon plays a leading role in the dynamic stabilisation of the distal radioulnar joint and the stabilisation of the ulnar side of the carpus. More specifically, it allows forearm rotation while the hand is lifting weight during the so-called loaded prono-supination tasks. Tendinopathies of the ECU are frequent in athletes using bats, clubs, or sticks. The level of stress undergone by the ECUt makes it prone to the risk of dislocation from its normal position [26]. The anatomic characteristics and fibrillar pattern of the canine ECUt suggest a vital role in stabilising the carpal joint during landing after a jump and during sharp turns, probably being stressed during agility tasks. Although no reports of injuries of the ECUt have been described in the veterinary literature, the authors have treated several sportive patients affected by chronic tendonitis responsible for lameness and decreased performance.

According to Williams et al., the primary role of the canine carpal flexor tendons (FCU, FDS, FDP, FCR) was to achieve stabilisation and oppose the standard extension at the carpus when the limb was in the stance phase of locomotion. During high-speed locomotion and jump landing, the carpal joint undergoes notable force, and the role played by the *Flexor Carpi Ulnaris* tendon is increased, opposing the hyperextension faced during agility tasks. Thoracic limb lameness due to FCU tendinopathies is reported in racing Greyhounds and lure-coursing dogs. A case report has suggested including FCU tendinopathy in the differential diagnosis for forelimb lameness in Weimaraners. Working and sporting patients seem prone to FCU tendon injuries due to the repeated stresses during performance, leading to chronic tendon strain injuries [27,28]. 

In canine sporting patients, traumatic injuries of the *Flexor Digitorum Superficialis* and *Profundus* tendons have been very well described. The clinical setting allows determining whether the FDS, the FDP, or both are involved. If the superficial flexor mechanism is damaged, the P1-P2 joint appears dropped with an excessive degree of flexion on manipulation. If the FDP tendon is also involved, the toenail will be seen off the ground due to its inability to flex P2-P3. The joint may also have increased extension on palpation. Repeated strain injuries followed by a very high weight-bearing load on the forelimbs during sporting activities may result in inflammation as the tendon attempts to heal. The result is the elongation of the tendon structure that may change the digit conformation (so-called “bowed tendons”), a mechanism abundantly described in equine athletes [27,29].

*Flexor Carpi Radialis* muscle in dogs contains more slow fibres in its medial compartment than the same muscle in other species; this suggests that it permits long walks. However, its lateral (humeral) compartment contains a more robust fast fibre population that is significant in carpal flexion during locomotion. Its role during the stance phase is to act as an antagonist of the flexor compartment, suggesting its importance during the landing part of the jump. Its involvement in sports-related dog injuries has not been reported [30].

The function of the canine *Abductor Pollicis Longus* tendon is to abduce the first digit, adduce the carpus, and stabilise it medially. The *Abductor Pollicis* groove is a common site for enthesophytes formation, and degenerative changes along its path are commonly reported in older dogs, especially Retrievers. According to previously published papers, its relationship with sports involving a repetitive carpal load, such as in agility dogs, can be logically assumed. The role of ultrasound in diagnosing APLt pathologies in dogs has previously been described. It has been considered helpful in classifying the differences between patients with diverse pathological findings, determining the extent of the APL tendon lesion, and scoring the grade of tendinitis or tenosynovitis [17,31].

## 5. Conclusions

According to recent veterinary sports medicine literature, many risk factors associated with agility training and competitions should be considered when referring to injuries. The age at which jump training begins is one of these factors. Since jumps are considered to be one of the most physiologically demanding tasks for agility dogs, the impact of this action on the forelimb should be considered [23]. Jumping has been defined as a demanding activity for dogs, leading to a high rate of injury [22]. Thus, the need to better understand the biomechanical principles of agility and athletic gesture becomes increasingly evident. A precise knowledge of anatomy can be considered to be a starting point in leading to the correct diagnosis. The role of US evaluation of the carpal tendons in sporting patients is promising and could lead to better understanding and treating agility-related injuries.

In conclusion, the anatomy and the ultrasonographic technique used to study the carpal tendons in sporting Border Collies are described in this paper. They could represent a starting point to better understand the role of chronic overuse of the carpal tendon in injuries of sporting patients.

## Figures and Tables

**Table 1:** The height and the length were obtained at the radial ulnar notch level in a transverse scan plan image of the tendon. The height was the short axis, and the length was the long axis of the tendon. The thickness was obtained in a longitudinal scan plan of the tendon at the same level and was the shot axis. All the measurements were made in mm.

Tendon		Right			Left	
	Height	Length	Thichness	Height	Length	Thichness
ECRt	1.16 ± 0.28	5.75 ± 1.07	1.11 ± 0.12	1.26 ± 0.21	5.72 ± 0.82	1.17 ± 0.13
EDCt	1.39 ± 0.12	6.62 ± 0.82	1.23 ± 1.28	1.50 ± 0.13	6.74 ± 0.89	1.28 ± 0.15
EDLt	0.95 ± 0.14	2.98 ± 0.21	0.95 ± 0.14	1.04 ± 0.15	3.04 ± 0.23	0.98 ± 0.11
ECULt	2.36 ± 0.17	4.54 ± 0.80	1.31 ± 0.19	2.35 ± 0.14	4.67 ± 0.81	1.37 ± 0.15
FCUt	1.79 ± 0.38	7.59 ± 1.16	2.04 ± 0.30	1.95 ± 0.43	7.81 ± 1.23	2.15 ± 0.31
FDSt	1.68 ± 0.30	6.54 ± 0.81	1.67 ± 0.20	1.82 ± 0.30	6.91 ± 0.38	1.75 ± 0.22
FDPt	1.96 ± 0.49	4.52 ± 0.84	1.79 ± 0.20	2.03 ± 0.45	4.78± 0.93	1.87 ± 0.23
FCRt	1.47 ± 0.42	2.64 ± 0.41	1.06 ± 0.32	1.55 ± 0.45	2.71 ± 0.34	1.14 ± 0.35
APLt	1.66 ± 0.17	4.01 ± 0.34	1.54 ± 0.25	1.75 ± 0.22	3.96 ± 0.41	1.58 ± 0.23

## Data Availability

Not applicable.

## Supplementary Materials

The following supporting information can be downloaded at: https://www.mdpi.com/article/10.3390/ani12162050/s1. Table S1. Signalment, tendon measurements, final diagnoses and reasons for excluding the carpus from the analysis are reported in the table.
