# Supplementary material for: High-Resolution Ultrasonographic Anatomy of the Carpal Tendons of Sporting Border Collies
Source: Animals (Basel). 2022 Aug 11;12(16):2050. doi: 10.3390/ani12162050 (PMC9404444; doi:10.3390/ani12162050)
Supplement: Supplementary file 1 [file animals-12-02050-s001.zip › animals-1840717-supplementary.pdf]

N	Kg	Sex	Age	Cr. face	Right			Left			Lat. face	Right			Left			Cd.Face	Right			Left			Med. Face	Right			Left			Main findings
					Height	Length	Thickness	Height	Length	Thickness		Height	Length	Thickness	Height	Lenght	Thickness		Height	Length	Thickness	Height	Length	Thickness		Height	Length	Thickness	Height	Length	Thickness	
1)	16,6	FS	50	ECRt	1,1	5,7	1	1,1	5,6	1,1	ECULt	2,4	4,6	1,3	2,3	4,5	1,4	FCUt	1,6	9,3	2,1	1,6	9,8	1,9	FCRt	1,4	3,4	0,7	1,3	3,2	0,8	Right infraspinatus tendon partial avulsion and right biceps tendon decreased diameter
				EDCt	1,6	7,1	1,2	1,7	8,9	1,1								FDSt	1,6	6,3	1,5	1,5	6,5	1,4	APLt	1,6	4,3	1,5	1,6	4,5	1,4	
				EDLt	1	3	0,9	0,9	2,9	0,9								FDPt	1,8	4,9	1,6	1,7	4,8	1,5								
2)	17,1	MI	20	ECRt	1	5,4	1,1	1,1	5,5	1	ECULt	2,5	4,8	1,2	2,4	4,9	1,3	FCUt	1,6	9,2	2,4	1,7	10,1	2,5	FCRt	1,5	3,1	0,9	1,6	3	0,9	Chronic right supraspinatus second-degree tendonitis with increased diameter, creating impingment
				EDCt	1,5	6,5	1,3	1,4	6,4	1,2								FDSt	1,8	6,6	1,6	1,7	6,5	1,7	APLt	1,6	4,2	1,6	1,6	4,2	1,4	syndrome on the right biceps brachii which shows mild diameter reduction
				EDLt	0,9	3,1	0,9	0,8	3,2	1								FDPt	1,8	5	1,6	1,8	4,9	1,6								
3)	16,9	MI	83	ECRt	0,9	5,2	1,1	1	5,3	1,2	ECULt	2,2	4,4	1,3	2,2	4,5	1,3	FCUt	1,6	9,1	2	1,6	9,9	1,9	FCRt	1,3	2,9	0,8	1,4	3,1	0,9	Right supraspinatus chronic second-degree tendonitis
				EDCt	1,4	6,3	1,2	1,5	6,5	1,2								FDSt	1,7	6,5	1,7	1,6	6,5	1,7	APLt	1,7	4,4	1,6	1,6	4,3	1,5	
				EDLt	0,9	2,9	0,8	1	2,8	1,1								FDPt	1,7	5,1	1,7	1,7	5	1,6								
4)	16	FS	75	ECRt	1,1	5,5	1				ECULt	2,3	4,3	1,2				FCUt	1,5	7,2	2,1				FCRt	1,3	3,1	1,1				Partial left biceps brachii tendon rupture, pain on left carpal flexion
				EDCt	1,3	6,1	1,2											FDSt	1,5	6,9	1,6				APLt	1,5	4,3	1,6				
				EDLt	0,9	2,8	0,9											FDPt	1,7	4,1	1,9											
5)	15,4	FS	63	ECRt	1,2	5,1	1,2	1,2	5,2	1,1	ECULt	2,1	4,2	1	2,2	4,3	1,1	FCUt	1,4	7	1,8	1,3	6,9	1,9	FCRt	1,1	2,7	0,7	0,9	2,7	0,8	Partial avulsion of the infraspinatus tendon
				EDCt	1,3	6,4	1,1	1,3	6,3	1,2								FDSt	1,5	6,4	1,6	1,6	6,6	1,5	APLt	1,5	4,1	1,5	1,4	4,3	1,5	
				EDLt	0,8	2,9	0,9	0,9	3	0,9								FDPt	1,7	4,2	1,8	1,6	4,4	1,8								
6)	15,8	MI	67	ECRt	1,1	5,3	1,1	1,2	5,4	1,1	ECULt	2,3	4,5	1,2	2,3	4,4	1,3	FCUt	1,8	7,1	2,3	1,7	7	1,8	FCRt	1,2	2,8	0,8	1,3	2,7	0,9	Chronic right supraspinatus second-degree tendonitis with increased diameter, creating impingment
				EDCt	1,4	6,5	1,2	1,4	6,4	1,2								FDSt	1,6	6,8	1,7	1,7	6,7	1,6	APLt	1,6	4	1,4	1,7	3,9	1,6	syndrome on the right biceps brachii which shows mild diameter reduction
				EDLt	1	3,1	1,1	0,9	3	1,1								FDPt	1,9	3,9	1,9	1,8	4,1	1,9								
7)	17,2	MI	115	ECRt	1,2	5,7	1,3	1,3	5,6	1,4	ECULt	2,4	2,6	1,3	2,3	2,7	1,4	FCUt	1,7	7,1	2,3	1,6	6,9	2,1	FCRt	1,2	2,5	1	1,3	2,4	0,9	Partial infraspunatus muscle rupture
				EDCt	1,5	6,3	1,4	1,6	6,4	1,4								FDSt	1,6	6,9	1,8	1,7	6,7	1,6	APLt	1,7	3,8	1,5	1,6	3,7	1,5	
				EDLt	1,1	3,2	1,1	1,2	3,3	1,1								FDPt	1,8	4,2	2,1	1,9	4,1	2								
8)	18,9	MI	95	ECRt				1,4	6,2	1,3	ECULt				2,3	5,1	1,5	FCUt				1,8	6,8	2,5	FCRt				1,3	2,6	0,8	Partial right biceps brachii tendon rupture, mild remodelling, and pain on flexion of the right carpus
				EDCt				1,6	6,7	1,5								FDSt				1,7	6,9	1,7	APLt				1,9	4,1	1,4	
				EDLt				1,2	3,3	1,1								FDPt				1,9	4	2								
9)	19,1	FS	97	ECRt				1,4	6,1	1,5	ECULt				2,5	4,9	1,4	FCUt				2,1	7,2	2,4	FCRt				1,4	2,6	1,1	Chronic right supraspinatus second-degree tendonitis with increased diameter, creating impingment
				EDCt				1,5	6,6	1,4								FDSt				1,8	7	1,9	APLt				2,1	4,3	1,7	syndrome on the right biceps brachii. Mild radiographic remodelling of the right carpus
				EDLt				1,1	3	1,1								FDPt				2	4,5	2,1								
10)	15,9	MI	49	ECRt	0,9	5,5	1,1				ECULt	2,2	3,8	1,3				FCUt	1,7	6,4	1,9				FCRt	1,3	2,4	1,1				Partial right biceps brachii tendon rupture. Pain on left carpus extension and flexion
				EDCt	1,4	6,2	1,2											FDSt	1,5	5,9	1,6				APLt	1,6	3,9	1,4				
				EDLt	0,9	2,7	1,1											FDPt	1,7	3,7	1,6											
11)	17	MI	71	ECRt	1,1	5,4	1,2	1,1	5,5	1,1	ECULt	2,3	4,4	1,4	2,2	4,6 1.3		FCUt	1,9	6,9	2,3	1,8	6,7	2,4	FCRt	1,2	2,3	0,7	1,1	2,4	0,8	Partial left biceps brachii tendon rupture
				EDCt	1,3	6,3	1,2	1,4	6,4	1,3								FDSt	1,7	6,7	1,6	1,6	6,9	1,5	APLt	1,7	3,2	1,7	1,8	3,3	1,6	
				EDLt	1	3	1,1	1,1	2,9	0,9								FDPt	1,7	3,8	1,8	1,7	3,9	1,7								
12)	16,8	MI	30	ECRt	0,8	5,3	1,1				ECULt	2,5	4,6	1,4				FCUt	1,7	6,5	1,9				FCRt	1,1	2,3	1				Chronic right supraspinatus second-degree tendonitis with increased diameter, creating impingment
				EDCt	1,4	6,3	1,3											FDSt	1,5	6,4	1,4				APLt	1,7	3,9	1,5				syndrome on right biceps brachii. Pain on left carpus flexion
				EDLt	0,9	2,7	1,1											FDPt	1,6	4,2	1,8											
13)	17,6	MC	93	ECRt	1,2	5,6	1,3				ECULt	2,5	4,7	1,5				FCUt	1,6	7,2	2,3				FCRt	1,4	2,7	1,2				Chronic right supraspinatus second-degree tendonitis with increased diameter, creating impingment
				EDCt	1,4	6,5	1,3											FDSt	1,6	7,1	1,7				APLt	1,8	4,3	1,5				syndrome on the right biceps brachii. Mild remodelling of the left carpus on radiographs
				EDLt	1	3,1	1											FDPt	1,9	3,8	1,9											
14)	20,8	MC	77	ECRt	1,7	9,1	1,2				ECULt	2,6	4,9	1,4				FCUt	1,6	9,9	2,4				FCRt	2,2	2,9	1,1				Partial right biceps brachii tendon rupture. Pain on left carpus extension and flexion
				EDCt	1,3	8,1	1,3											FDSt	1,2	4,6	1,3				APLt	2	4,6	1,6				
				EDLt	1,2	3,4	0,7											FDPt	3,2	5,3	2											
15)	18,1	FS	75	ECRt				1,5	5,2	1,3	ECULt				2,4	4,6	1,5	FCUt				1,7	6,9	2,3	FCRt				1,2	2,7	1	Chronic right supraspinatus second-degree tendonitis with increased diameter, creating impingment
				EDCt				1,5	6,7	1,4								FDSt				1,5	6,4	1,6	APLt				1,8	3,9	1,5	syndrome on the right biceps brachii which shows mild diameter reduction. Pain on right carpal flexion
				ECRt				1,1	3,2	0,9								FDPt				1,9	4,3	1,8								
16)	17,1	FS	92	ECRt	1,7	5,1	1,2	1,6	5,2	1,3	ECULt	2,1	4,7	1,3	2,1	4,7	1,2	FCUt	2,4	6,2	1,9	2,4	6,4	2,1	FCRt	1,5	2,5	0,9	1,4	2,2	1,1	Left elbow moderate OA
				EDCt	1,3	6,2	1,3	1,4	6,1	1,2								FDSt	2,2	7	1,9	2,3	7,2	1,8	APLt	1,8	3,9	1,2	1,9	4	1,3	
				EDLt	1	2,9	0,8	1,1	3	0,8								FDPt	2,1	4,6	1,6	2,2	4,4	1,7								
17)	20,9	FS	42	ECRt	1,7	8,6	1,1	1,6	8,7	1,2	ECULt	2,8	7,3	1,8	2,6	7,2	1,7	FCUt	2,6	9,3	2,1	2,5	9,1	2,2	FCRt	2,2	2,8	1,6	2,3	2,6	1,5	Partial avulsion of the right triceps tendon
				EDCt	1,6	9,6	1,4	1,7	9,5	1,5								FDSt	2,2	7,2	1,9	2,3	7,3	1,9	APLt	1,7	4,1	1,9	1,8	4,2	1,8	
				EDLt	1,3	3,3	1,1	1,3	3,4	1,2								FDPt	3,3	6,8	2,3	3,2	6,8	2,2								

18)	18,8	MI	133	ECRt	1,1	5,6	1,2	1,2	5,4	1,1	ECULt	2,2	4,9	1,4	2,3	4,7	1,4	FCUt	2,5	8,1	2,3	2,6	8,3	2,4	FCRt	2,5	3,1	1,7	2,4	3,2	1,7	Chronic right supraspinatus second-degree tendonitis with increased diameter, creating impingment
				EDCt	1,4	6,3	1,3	1,4	6,4	1,2								FDSt	2,4	7,6	2,1	2,3	7,6	2,2	APLt	1,9	4,3	2,2	2	4,2	2,1	syndrome on the left biceps
				EDLt	1	3,1	0,9	1,1	2,9	0,9								FDPt	2,3	5,9	1,9	2,4	6,1	2,3								
19)	15,9	FS	62	ECRt	1	5,4	1,1	1,1	5,3	1,1	ECULt	2,3	4,3	1,3	2,2	4,2	1,3	FCUt	1,8	7	1,4	1,7	7,1	1,3	FCRt	1,3	2,5	1,2	1,4	2,4	1,1	Partial diameter reduction of the left biceps brachii tendon
				EDCt	1,3	6,3	1,1	1,4	6,3	1,2								FDSt	1,6	6,5	1,6	1,7	6,5	1,6	APLt	1,5	3,8	1,3	1,4	3,9	1,3	
				EDLt	0,9	2,9	1	1	3	0,9								FDPt	1,8	3,8	1,7	1,8	3,7	1,8								
20)	18,2	MC	79	ECRt	1,2	5,5	1,2	1,3	5,4	1,1	ECULt	2,4	4,7	1,3	2,4	4,6	1,3	FCUt	1,9	7,5	2,2	2	7,5	2,4	FCRt	1,7	2,8	1,5	1,7	2,7	1,5	Partial rupture of the left brachial muscle
				EDCt	1,4	6,7	1,4	1,5	6,5	1,4								FDSt	1,7	6,9	1,7	1,6	7	1,7	APLt	1,8	4	1,8	1,7	4,1	1,7	
				EDLt	1	2,9	1	1,1	3	0,9								FDPt	2,1	5,1	1,7	2,2	5,1	1,8								
21)	14,3	FS	99	ECRt	0,8	4,9	0,9	0,8	5	1	ECULt	2,3	4,1	1	2,4	4	1,1	FCUt	1,4	6,6	1,8	1,4	6,5	1,8	FCRt	1,1	1,9	0,7	1,1	2	0,8	Severe left biceps brachii tendon sheath effusion and increased thickness
				EDCt	1,2	6	1	1,3	6,1	0,9								FDSt	1,4	6,9	1,5	1,5	6,8	1,7	APLt	1,5	3,2	1,3	1,4	3,1	1,2	
				EDLt	0,7	2,5	0,8	0,7	2,4	0,9								FDPt	1,6	3,8	1,6	1,5	3,8	1,6								
22)	14,6	FS	45	ECRt	1	5,2	1				ECULt	2,4	4,4	1,2				FCUt	1,5	6,6	1,6				FCRt	1,2	2,1	1				Partial right biceps brachii tendon rupture. Thickened and less defined left APLt
				EDCt	1,3	6,1	1,1											FDSt	1,5	4,2	1,6				APLt	1,4	3,4	1,2				
				EDLt	0,8	3	0,9											FDPt														
23)	18,9	MI	100	ECRt				1,2	5,5	1,1	ECULt				2,6	5,1	1,5	FCUt				2,6	8,1	2,3	FCRt				2,2	3,2	1,7	Chronic right supraspinatus second-degree tendonitis with increased diameter, creating impingment
				EDCt				1,6	6,6	1,3								FDSt				2,3	7,3	1,9	APLt				2,1	4	1,9	syndrome on the left biceps. Pain on left carpus flexion
				EDLt				1,1	3,3	1								FDPt				2,3	5,8	2,1								
24)	19,2	MC	38	ECRt				1,3	6,1	1,2	ECULt				2,5	4,9	1,5	FCUt				2,5	8,6	2,3	FCRt				2,1	2,9	1,6	Partial left biceps brachii tendon rupture. Thickening and loss of fibrillar pattern of the right
				EDCt				1,5	6,8	1,4								FDSt				2,1	7,4	2	APLt				1,9	4,1	1,8	flexor digitorum superficialis tendon.
				ECRt				1,2	3	1								FDPt				2,9	6,4	2,1								
25)	14,2	FS	53	ECRt	0,9	5,3	0,8				ECULt	2,2	4,3	1,1				FCUt	1,4	6,5	1,5				FCRt	1,1	1,8	0,8				Chronic right supraspinatus second-degree tendonitis with increased diameter, creating impingment
				EDCt	1,2	6,4	1											FDSt	1,4	6,6	1,5				APLt	1,3	3,2	1,2				syndrome on the right biceps brachii. Pain in left carpus extension and flexion
				EDLt	0,7	2,8	0,7											FDPt	1,5	3,9	1,5											
26)	19,3	MI	85	ECRt	1,5	6,3	1,2	1,5	6,4	1,1	ECULt	2,5	4,8	1,6	2,4	4,9	1,5	FCUt	2,5	8,6	2,3	2,5	8,7	2,3	FCRt	2,1	2,9	1,7	2,1	2,9	1,7	Left brachiocephalic and deltoideus myositis
				EDCt	1,6	6,8	1,4	1,7	6,5	1,3								FDSt	2,1	7,4	2,1	2,1	7,5	2,2	APLt	1,9	4,2	1,8	1,9	4,1	1,8	
				EDLt	1	3,2	1,1	1,1	3,1	1								FDPt														
